# Preoperative serum inflammatory markers in the prognostic assessment of hepatocellular carcinoma resection in stages I/II

**DOI:** 10.3389/fmolb.2025.1640390

**Published:** 2025-08-15

**Authors:** Fan Liu, Yuan Xiang, Hao Xu, Xiaoxue Xu

**Affiliations:** ^1^ Department of Radiology, Affiliated Hospital of North Sichuan Medical College, Nanchong, Sichuan, China; ^2^ Department of Obstetrics, Affiliated Hospital of North Sichuan Medical College, Nanchong, Sichuan, China

**Keywords:** hepatocellular carcinoma, serum inflammatory markers, prognostic assessment, nomogram, overall survival

## Abstract

**Background:**

Hepatocellular carcinoma (HCC) remains a global health challenge, with early-stage resection offering the best chance for improved outcomes. However, limitations of the TNM staging system highlight the need for additional prognostic tools. This study evaluates the prognostic value of preoperative serum inflammatory markers in patients with stage I/II HCC undergoing surgical resection.

**Methods:**

A retrospective cohort study was conducted on 410 HCC patients (stage I/II) who underwent surgical resection at the Affiliated Hospital of North Sichuan Medical College between November 2011 and March 2020. Clinical and serological data, including neutrophil-to-lymphocyte ratio (NLR), platelet-to-lymphocyte ratio (PLR), and platelet-to-neutrophil ratio (PNR), were analyzed. Prognostic factors for overall survival (OS) were identified through univariate and multivariate Cox regression analyses. A nomogram was developed to predict 1-year, 3-year, and 5-year OS, with its performance assessed using ROC curves, calibration plots, and decision curve analysis (DCA). Kaplan-Meier survival curves were used to compare risk groups, and the model’s predictive efficacy was evaluated against the AJCC 8th Edition TNM staging system.

**Results:**

Multivariate Cox regression identified NLR, PLR, ALBI score, AFP levels, and HBeAg status as independent prognostic factors for OS. The nomogram demonstrated superior discriminatory power (AUC: 0.78, 0.74, and 0.71 for 1-, 3-, and 5-year OS, respectively) compared to TNM staging. Kaplan-Meier analysis revealed significantly worse OS in the high-risk group (log-rank *p* < 0.001). The nomogram outperformed the AJCC TNM system in both discrimination and clinical utility, as validated by decision curve analysis and the Integrated Discrimination Improvement Index.

**Conclusion:**

Preoperative serum inflammatory markers, when integrated with traditional TNM staging, significantly improve prognostic accuracy for stage I/II HCC patients undergoing surgical resection. The developed nomogram provides a practical tool for individualized risk stratification and may guide postoperative management to improve patient outcomes.

## Graphical Abstract

**Figure:**
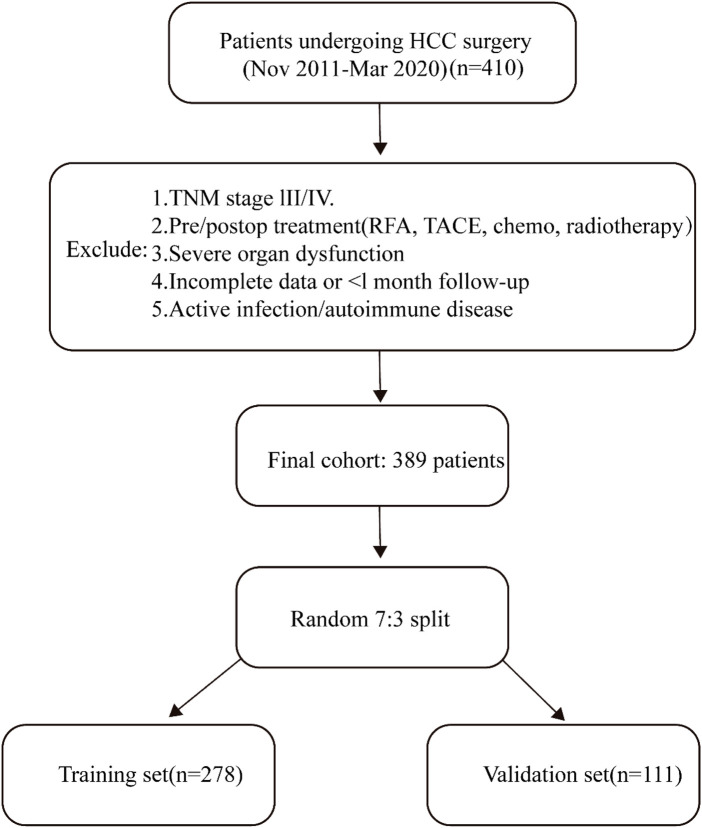
Research Flowchart.

## 1 Introduction

Hepatocellular carcinoma (HCC) remains a global health challenge with significant variability in incidence and mortality rates across different regions (Parra-Herran et al., 2021). This highlights the need for region-specific strategies in addressing HCC. Recent studies underscore the crucial importance of early-stage diagnosis and treatment, particularly emphasizing the role of surgical resection in improving patient outcomes (Singal et al., 2022; Ducreux et al., 2023). Early postoperative intervention may improve the prognosis of patients.

Traditional TNM is widely used in clinical practice, but with continuous progress in medical technology, it is increasingly recognized that TNM alone may not fully capture the biological heterogeneity of HCC (Lea et al., 2014). A growing number of studies have noted the need to link biomarkers to traditional TNM (Balch et al., 2003; Zheng et al., 2023). Serological inflammatory markers—such as C-reactive protein (CRP), neutrophil-to-lymphocyte ratio (NLR), platelet-lymphocyte ratio (PLR), gamma-glutamyl transferase (GGT), and carbohydrate antigens (CA199, CA125)—reflect the host’s inflammatory and immune response, which plays a crucial role in tumor initiation, progression, and metastasis (McMillan, 2009; Guthrie et al., 2013; Templeton et al., 2014a; Liu et al., 2019; Zhang et al., 2019). Chronic inflammation can promote angiogenesis, inhibit apoptosis, and facilitate tumor cell invasion, all of which affect prognosis. These markers offer a non-invasive means to assess patient prognosis. Emerging evidence suggests their relevance in HCC prognosis, paving the way for more refined prognostic evaluations (Zhang et al., 2023). Integrating these markers into clinical practice helps improve prognostic accuracy and can inform treatment decisions (Giannone et al., 2023).

Despite advancements, current prognostic assessment methods for HCC, including imaging and tumor markers, have notable limitations (Jiang et al., 2013; Ahn et al., 2021), such as the inability to detect microscopic tumor features (e.g., microvascular invasion) or fully reflect biological heterogeneity. In addition, tumor markers like AFP lack sufficient sensitivity and specificity in a subset of patients. These limitations can lead to suboptimal treatment strategies. Addressing these challenges is vital for the development of more reliable prognostic tools (Ahn et al., 2021). Thus, integrating inflammatory markers may help overcome these shortcomings and guide more accurate risk stratification and treatment strategies.

The objective of this study was to evaluate the prognostic value of preoperative serological inflammatory markers in patients with stage I/II HCC based on traditional TNM staging. Understanding the predictive power of these markers could transform patient care. By understanding the role of these markers, this study provides a basis for postoperative management of liver cancer and the development of personalized treatment plans with a view to improving clinical outcomes.

## 2 Methods

### 2.1 Patient Selection

A retrospective search was conducted on the clinical data of liver cancer patients who underwent surgical treatment at the Affiliated Hospital of North Sichuan Medical College from November 2011 to March 2020. Inclusion criteria: Patients with a first-time pathological or clinical diagnosis of HCC; TNM staging classified as stage I or II; preoperative liver reserve function classified as Child-Pugh grade A or B. Exclusion criteria: Patients with severe brain, heart, lung, or kidney dysfunction at the time of first diagnosis; those who underwent radiofrequency ablation, transarterial chemoembolization, chemotherapy, or radiotherapy before or concurrently with surgery; TNM staging classified as stage III/IV; patients with incomplete clinical and follow-up information, or with a follow-up period of less than 1 month. Patients with active infections or other chronic inflammatory or autoimmune conditions at the time of surgery were also excluded to minimize confounding effects on systemic inflammatory markers. A total of 389 patients met the inclusion criteria and were included in the final analysis. Patients were randomly divided into training and validation sets in a 7:3 ratio, a commonly adopted split in predictive modeling literature to balance model stability and evaluation (Wen et al., 2022; He et al., 2023). All surgeries were performed by experienced attending or senior hepatobiliary surgeons following standardized institutional protocols, which helped maintain consistency in surgical technique throughout the study period. This retrospective study was approved by the Ethics Committee of the Affiliated Hospital of North Sichuan Medical College (Approval File Number: 2024ER665-1). Due to the retrospective nature of the study, the Ethics Committee waived the requirement for informed consent.

### 2.2 Indicators

Research indicators were obtained from the patients’ clinical data, which include age, gender, neutrophil-to-lymphocyte ratio (NLR), platelet-to-lymphocyte ratio (PLR), platelet-to-neutrophil ratio (PNR), prothrombin time (PT), activated partial thromboplastin time (APTT), fibrinogen (FIB), thrombin time (TT), globulin (GLB), Albumin-Bilirubin (ALBI) score: (log10 bilirubin × 0.66) + [albumin × (−0.085)], aspartate aminotransferase (AST), alanine aminotransferase (ALT), gamma-glutamyl transpeptidase (GGT), alpha-L-fucosidase (AFU), hepatitis B e antigen (HBeAg), high-precision hepatitis B virus DNA, alpha-fetoprotein (AFP), carbohydrate antigen 125 (CA125), and TNM staging. All laboratory values were obtained within 1 week prior to surgery as part of routine preoperative testing. The measurements were performed using standardized automated analyzers in the hospital’s central laboratory, following manufacturer protocols. These indicators were all recorded before surgery.

Notably, indicators such as hepatitis C virus (HCV) infection status, protein induced by vitamin K absence-II (PIVKA-II), and Model for End-Stage Liver Disease (MELD) score were not included in the analysis. This was primarily due to incomplete or inconsistent data availability across the study period, as these indicators were not part of the standard preoperative workup during earlier years of the data collection (2011–2020). Therefore, to ensure data quality and comparability, only routinely available and complete indicators were included.

### 2.3 Follow-up

Typically, patients were followed up every 6 months for the first 2 years after the diagnosis of liver cancer, and then annually thereafter, to obtain information on the patients’ treatment and living conditions. Follow-up data were collected through regular outpatient visits and structured telephone interviews conducted by trained clinical staff. The last follow-up took place in March 2021. Overall survival (OS) was defined as the time from the date of surgery to death or last follow-up.

### 2.4 Statistics

Statistical analyses were conducted using SPSS 27 and R 4.3.1 software, with the X-tile software employed to convert continuous variables into categorical variables. Continuous variables that were normally distributed are represented as (x̄ \pm SD \). Continuous variables not normally distributed are represented as the median (1st quartile - 3rd quartile). Categorical variables are expressed as frequencies and percentages (n/%). In the training set, variables with a univariate Cox regression analysis *P*-value less than 0.05 were included in the multivariate Cox regression analysis, using the Akaike Information Criterion for stepwise backward selection to determine the independent risk factors affecting OS. This stepwise procedure was applied to reduce overfitting and eliminate multicollinearity among predictors. These factors were then introduced into the R software to construct a nomogram for predicting 1-year, 3-year, and 5-year OS after hepatectomy for liver cancer, using the area under the ROC curve (AUC) to assess the discriminatory power of the nomogram, the calibration correction curve to evaluate its calibration, and decision curve analysis (DCA) to assess the clinical utility of the nomogram. The predictive performance and clinical efficacy of the nomogram were validated using the validation set data. The model was used to calculate the prognostic risk score for each patient, and the median risk score was used as the cutoff to divide patients into high-risk and low-risk groups. Survival curves were plotted using the Kaplan-Meier method, and the log-rank test was used. The Integrated Discrimination Improvement Index (IDI) was used to compare the predictive power of the two models. To further ensure the stability and robustness of the model, internal validation was conducted using bootstrapping with 1,000 resamples during model development. In all analyses, a *P*-value less than 0.05 was considered to indicate a statistically significant difference.

## 3 Results

### 3.1 Patient general clinical data

A total of 410 liver cancer patients were initially identified. After applying exclusion criteria (e.g., advanced-stage disease, incomplete clinical data, missing follow-up, or confounding inflammatory conditions), 389 patients were included in the final analysis. These patients were randomly divided into a training set and a validation set in a 7:3 ratio. The clinical baseline characteristics of the two cohorts are shown in Table 1. The training set and validation set consisted of 278 and 111 cases, respectively, with median survival times of 79 months and 73 months, and median follow-up times of 53 months and 54 months, respectively. There were no significant differences in the baseline characteristics between the two groups (*P* > 0.05). Factors related to liver function status—such as albumin, bilirubin, ALBI score, and liver enzymes (ALT, AST, GGT)—were included as baseline variables and showed balanced distributions between the two groups. Moreover, all included patients had preoperative liver reserve function of Child-Pugh class A or B.

**TABLE 1:** Patient demographics and clinical characteristics.

	Training set (n = 278)	Validation set (n = 111)	*P*
gender, n (%)			0.856
female	65 (23.38)	25 (22.52)	
male	213 (76.62)	86 (77.48)	
Tumor stage, n (%)			0.445
T1	167 (60.07)	62 (55.86)	
T2	111 (39.93)	49 (44.14)	
Node stage, n (%)			1.000
N0	278 (100.00)	111 (100.00)	
N1			
Metastasis stage, n (%)			1.000
M0	278 (100.00)	111 (100.00)	
M1			
HBeAg, n (%)			0.354
Positive	51 (18.35)	16 (14.41)	
Negative	227 (81.65)	95 (85.59)	
Age, mean (±SD)	56.95 ± 10.83	57.55 ± 9.98	0.615
PLT, median [IQR]	140.00 [96.00, 194.00]	146.00 [106.00, 195.00]	0.302
Lym, mean (±SD)	30.54 ± 11.08	28.34 ± 10.84	0.077
Neu, median [IQR]	58.40 [50.40, 66.50]	59.70 [53.50, 66.20]	0.276
PNR, median [IQR]	2.42 [1.70, 3.49]	2.46 [1.84, 3.30]	0.847
PLR, median [IQR]	4.70 [3.10, 7.30]	5.20 [3.60, 8.30]	0.084
NLR, median [IQR]	1.96 [1.36, 2.87]	2.16 [1.53, 2.92]	0.348
PT, median [IQR]	12.60 [11.90, 13.60]	12.50 [11.80, 13.20]	0.356
APTT, median [IQR]	28.50 [26.40, 31.40]	28.20 [26.30, 30.50]	0.641
FIB, median [IQR]	2.23 [1.87, 2.70]	2.21 [1.88, 2.73]	0.890
TT, median [IQR]	19.20 [18.30, 20.60]	19.10 [18.10, 20.20]	0.365
GLB, median [IQR]	28.50 [25.30, 31.90]	27.99 [24.98, 31.37]	0.515
ALB, median [IQR]	40.00 [35.60, 43.60]	40.50 [37.80, 42.70]	0.446
TBIL, median [IQR]	16.70 [12.20, 23.20]	16.80 [11.50, 23.00]	0.698
ALBI, median [IQR]	−2.61 [-2.96, -2.24]	−2.63 [-2.91, -2.41]	0.510
ALT, median [IQR]	30.00 [18.50, 51.00]	31.00 [21.00, 47.00]	0.537
AST, median [IQR]	30.00 [22.00, 47.00]	32.90 [23.00, 51.00]	0.566
GGT, median [IQR]	49.00 [31.00, 90.00]	53.00 [36.00, 93.00]	0.434
AFU, median [IQR]	26.00 [21.00, 31.00]	26.00 [21.00, 31.10]	0.972
DNA, median [IQR]	0.00 [0.00, 23100.00]	0.00 [0.00, 7540.00]	0.828
CEA, median [IQR]	2.55 [1.65, 3.85]	2.80 [1.90, 4.14]	0.156
AFP, median [IQR]	20.00 [4.10, 270.00]	24.00 [6.30, 540.00]	0.176
CA199, median [IQR]	9.49 [4.77, 19.02]	11.51 [4.97, 20.78]	0.540
CA125, median [IQR]	13.30 [9.40, 21.60]	12.60 [8.50, 18.00]	0.145
mOS	79.00 [59.54, 98.46]	73.00 [58.93, 87.07]	0.605
median follow-up time	53.00 [46.96, 59.04]	54.00 [45.88, 62.12]	0.334

Note: Values are presented as mean ± SD, or median [IQR] unless otherwise indicated. Units—Age: years; PLT (platelet count): ×10^9^/L; Neu (neutrophils), Lym (lymphocytes): %; PT (prothrombin time), APTT (activated partial thromboplastin time), TT (thrombin time): seconds; FIB (fibrinogen): g/L; ALB, GLB (albumin, globulin): g/L; TBIL (total bilirubin): μmol/L; ALT, AST, GGT (liver enzymes): U/L; AFP, CA199, CA125, CEA: ng/mL; AFU: U/L; HBV-DNA: IU/mL. Abbreviations—NLR, neutrophil-to-lymphocyte ratio; PLR, platelet-to-lymphocyte ratio; PNR, platelet-to-neutrophil ratio; ALBI, albumin-bilirubin score; SD, standard deviation; IQR, interquartile range.

### 3.2 Optimal cutoff values for continuous variables and variable transformation

To more clearly demonstrate the relationship between study variables and outcome variables, this study converted continuous variables into categorical ones. The X-tile software was used to calculate the optimal clinical cutoff values, and these cutoffs were used to categorize the continuous variables. For example, the cutoff for age was 71 years, dividing all patients into two groups: ≤71 and >71. This method was used to convert all continuous variables into categorical variables, which were then represented as percentages, as shown in Table 2.

**TABLE 2:** Categorization of continuous variables using X-tile software.

	Training set (n = 278)	Validation set (n = 111)	*P*
age, n (%)			
≤71	249 (89.57)	103 (92.79)	0.328
>71	29 (10.43)	8 (7.21)	
PLR, n (%)			
>8.56	49 (17.63)	26 (23.42)	0.191
≤8.56	229 (82.37)	85 (76.58)	
PNR, n (%)			
>1.9	187 (67.27)	80 (72.07)	0.356
≤1.9	91 (32.73)	31 (27.93)	
NLR, n (%)			
≤2.17	153 (55.04)	56 (50.45)	0.413
>2.17	125 (44.96)	55 (49.55)	
PT, n (%)			
>12.9	111 (39.93)	35 (31.53)	0.122
≤12.9	167 (60.07)	76 (68.47)	
APTT, n (%)			
≤34.1	241 (86.69)	96 (86.49)	0.957
>34.1	37 (13.31)	15 (13.51)	
FIB, n (%)			
≤2.95	232 (83.45)	93 (83.78)	0.937
>2.95	46 (16.55)	18 (16.22)	
TT, n (%)			
>21.6	39 (14.03)	10 (9.01)	0.178
≤21.6	239 (85.97)	101 (90.99)	
GLB, n (%)			
>30.5	96 (34.53)	34 (30.63)	0.461
≤30.5	182 (65.47)	77 (69.37)	
ALBI, n (%)			
>-1.39	13 (4.68)	2 (1.80)	0.354
−2.60–1.39	125 (44.96)	48 (43.24)	
≤-2.60	140 (50.36)	61 (54.95)	
ALT, n (%)			
≤80	206 (74.10)	78 (70.27)	0.442
>80	72 (25.90)	33 (29.73)	
AST, n (%)			
≤53.2	222 (79.86)	85 (76.58)	0.474
>53.2	56 (20.14)	26 (23.42)	
GGT, n (%)			
≤22.6	97 (34.89)	34 (30.63)	0.422
>22.6	181 (65.11)	77 (69.37)	
AFU, n (%)			
≤30	207 (74.46)	79 (71.17)	0.507
>30	71 (25.54)	32 (28.83)	
DNA, n (%)			
597–435000	74 (26.62)	29 (26.13)	0.877
≤597	174 (62.59)	68 (61.26)	
>435, 000	30 (10.79)	14 (12.61)	
CEA, n (%)			
≤5.92	261 (93.88)	94 (84.68)	0.004
>5.92	17 (6.12)	17 (15.32)	
AFP, n (%)			
≤837.8	246 (88.49)	87 (78.38)	0.010
>837.8	32 (11.51)	24 (21.62)	
CA199, n (%)			
≤31.4	244 (87.77)	101 (90.99)	0.365
>31.4	34 (12.23)	10 (9.01)	
CA125, n (%)			
≤32.1	230 (82.73)	99 (89.19)	0.111
>32.1	48 (17.27)	12 (10.81)	

Note: Cutoff values were determined using X-tile software. Units and abbreviations are the same as in Table 1.

### 3.3 Identification of prognostic factors for postoperative survival in patients

In the training set, univariate analysis identified Tumor stage, PT, APTT, TT (Thrombin Time), AST, GGT, CA199, CA125, NLR, GLB, PNR, and ALBI as potential risk factors affecting the postoperative survival period of liver cancer patients. Although age was included as a candidate variable, it did not show a statistically significant association with OS in univariate analysis (*P* > 0.05) and was thus not retained in multivariate modeling. Multivariate analysis incorporating these variables revealed that Tumor stage, APTT, GGT, CA199, CA125, and PNR are independent risk factors for the postoperative survival period of liver cancer patients. Detailed analysis results are presented in Table 3.

**TABLE 3:** Univariate and multivariate cox hazards analysis of the Training cohort.

	Univariate analysis		Multivariate analysis	
	HR [95% CI]	*P*	HR [95% CI]	*P*
gender				
female vs. male	1.08 [0.65, 1.74]	0.745		
age				
>71 vs. ≤71	1.39 [0.75, 2.55]	0.294		
Tumorstage				
T2 vs. T1	2.54 [1.68, 3.85]	<0.001	2.10 [1.37, 3.22]	0.001
Nodestage				
N1 vs. N0				
Metastasisstage				
M1 vs. M0				
NLR				
>2.17 vs. ≤2.17	1.55 [1.03, 2.35]	0.036		
GLB				
>30.5 vs.≤30.5	2.18 [1.45, 3.30]	<0.001		
PNR				
>1.9 vs. ≤1.9	0.45 [0.30, 0.67]	<0.001	0.59 [0.38, 0.91]	0.018
PLR				
>8.56 vs. ≤8.56	0.91 [0.52, 1.58]	0.735		
ALBI				
>-2.6-1.39 vs. ≤30.5	1.49 [0.97, 2.30]	0.069		
>-1.39 vs.≤30.5	3.08 [1.48, 6.39]	0.003		
PT				
>21.6 vs. ≤21.6	2.66 [1.75, 4.06]	<0.001		
APTT				
>34.1 vs. ≤34.1	2.78 [1.75, 4.44]	<0.001	2.71 [1.66, 4.43]	<0.001
FIB				
>2.95 vs. ≤2.95	1.53 [0.92, 2.57]	0.105		
TT				
>21.6 vs. ≤21.6	1.93 [1.20, 3.10]	0.007		
ALT				
>22.6 vs. ≤22.6	1.52 [0.96, 2.42]	0.078		
AST				
>53.2 vs. ≤53.2	2.37 [1.52, 3.71]	<0.001		
GGT				
>80 vs. ≤80	3.55 [2.33, 5.40]	<0.001	2.51 [1.57, 4.03]	<0.001
AFU				
>30 vs. ≤30	1.24 [0.80, 1.94]	0.337		
HBeAg				
Positive vs. Negative	1.37 [0.85, 2.21]	0.203		
DNA				
597–435000 vs.≤597	1.34 [0.85, 2.14]	0.211		
>435, 000 vs.≤597	1.70 [0.94, 3.07]	0.081		
CEA				
>5.92 vs. ≤5.92	1.37 [0.60, 3.13]	0.461		
AFP				
>837.8 vs. ≤837.8	1.41 [0.79, 2.54]	0.247		
CA199				
1	3.30 [2.06, 5.27]	<0.001	1.88 [1.15, 3.09]	0.012
CA125				
>32.1 vs. ≤32.1	3.33 [2.12, 5.21]	<0.001	2.53 [1.57, 4.05]	<0.001

Note: HR, hazard ratio; CI, confidence interval. All abbreviations are defined in Table 1.

### 3.4 Nomogram construction and validation

**FIGURE 1:**
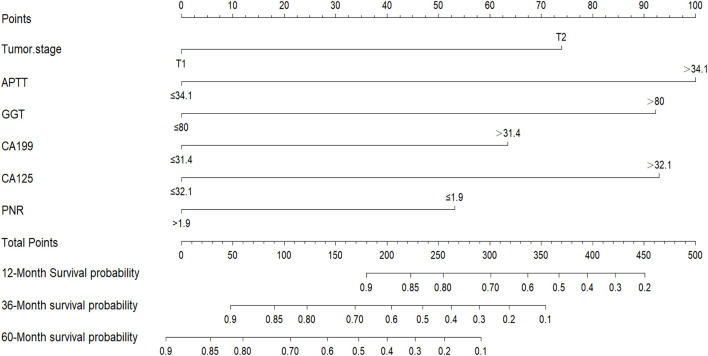
Nomogram for predicting the survival of patients undergoing surgery for stage I/II.

**FIGURE 2:**
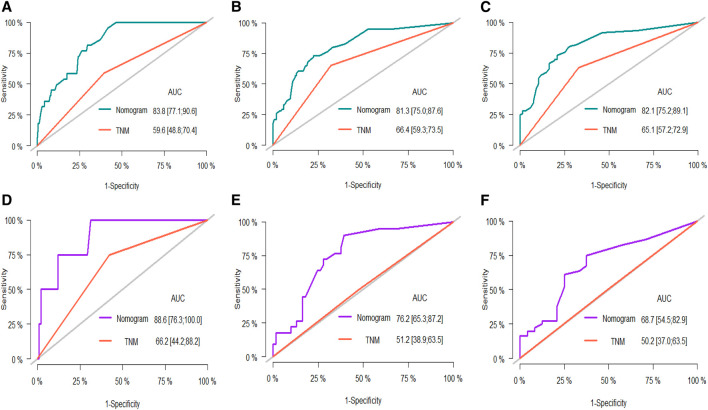
ROC curves for the nomogram in the training set at 1, 3, and 5 years: **(A–C)** ROC curves in the validation set at 1, 3, and 5 years: **(D–F)**.

**FIGURE 3:**
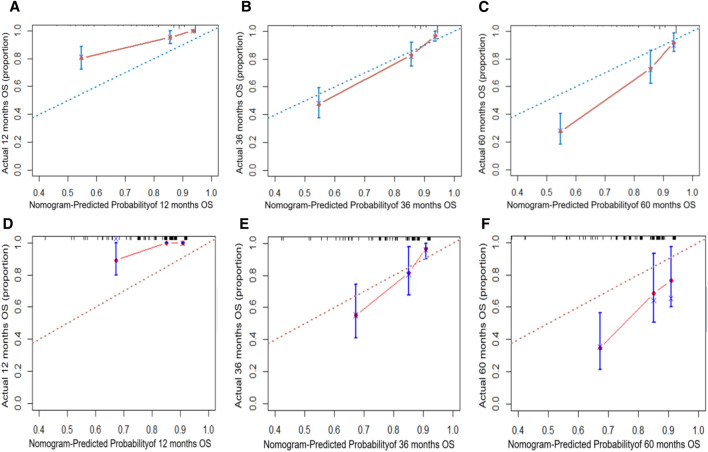
Calibration curves for the nomogram in the training set at 1, 3, and 5 years: **(A–C)** calibration curves in the validation set at 1, 3, and 5 years: **(D–F)**.

The independent risk factors identified by the multivariate Cox analysis were incorporated into the R software to establish a nomogram for predicting the 1-, 3-, and 5-year overall survival (OS) of patients with stage I/II liver cancer after surgery (Figure 1). In the training set, the area under the ROC curve (AUC) for 1, 3, and 5 years was 83.8%, 81.3%, and 82.1%, respectively, and in the validation set, it was 88.6%, 76.2%, and 68.7%, respectively (Figures 2A–F), demonstrating the model’s discriminatory performance. Figures 3A–F show that the calibration curves for the training and validation sets at 1, 3, and 5°years all demonstrate a good consistency between the predicted probabilities and the actual occurrence probabilities.

### 3.5 Comparison of the nomogram with the AJCC 8th edition TNM staging system

**FIGURE 4:**
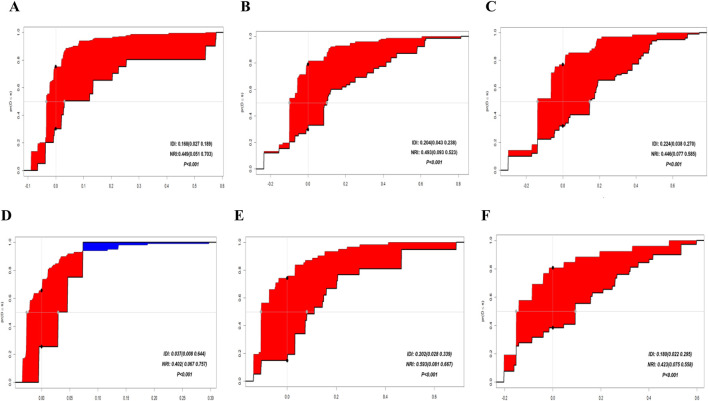
IDI curves for the training set at 1, 3, and 5 years: **(A–C)** IDI curves for the validation set at 1, 3, and 5 years: **(D–F)**.

**FIGURE 5:**
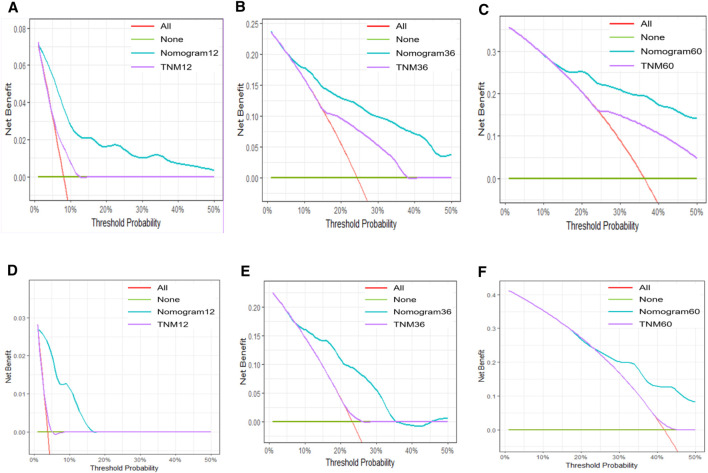
DCA curves for the nomogram in the training set at 1, 3, and 5°years: **(A–C)**; DCA curves in the validation set at 1, 3, and 5°years: **(D–F)**.

In both the training and validation cohorts, the AUCs of the nomogram were higher than those of the AJCC 8th Edition TNM staging system (Figures 2A–F). IDI values for 1-, 3-, and 5-year OS were all greater than 0 (Figure 4). Decision curve analysis (Figure 5) showed that the net benefit of the nomogram was greater than that of the TNM system across relevant threshold probabilities in both cohorts.

### 3.6 Kaplan-Meier Survival Curves

**FIGURE 6:**
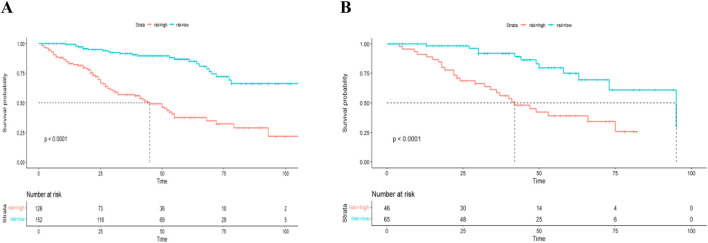
Kaplan-Meier survival curves for the nomogram: **(A)** in the training set, **(B)** in the validation set.

Based on the nomogram-derived risk score, patients were stratified into high- and low-risk groups using the median value of 92.26. In the training set, the median OS was 45 months in the high-risk group, while in the low-risk group, the cumulative survival rate remained above 50% during follow-up, and median OS could not be estimated. In the validation set, median OS was 42 months in the high-risk group and 95 months in the low-risk group. Kaplan–Meier survival analysis showed significantly better survival in the low-risk group compared to the high-risk group (*P* < 0.001; Figure 6).

## 4 Discussion

The main findings of this study were to explore the prognostic significance of the traditional TNM basis combined with preoperative serological inflammatory markers such as APTT, GGT, CA199, CA125, and PNR in stage I/II HCC. These indicators have shown substantial predictive value for postoperative prognosis. By incorporating these independent risk factors, determined through multi-factor Cox analysis, into R software, we constructed a nomogram to predict overall survival (OS) for patients 1, 3, and 5 years post-surgery (He et al., 2023). The predictive performance of this model was demonstrated by the area under the ROC curve (AUC), which was 83.8%, 81.3%, and 82.1% in the training set for 1, 3, and 5 years, respectively, and 88.6%, 76.2%, and 68.7% in the verification set. These results indicate good model differentiation. Additionally, the calibration curves for both sets showed excellent agreement between predicted and actual probabilities, suggesting robust calibration.

The findings from this study have significant clinical implications. The identified serological inflammatory markers and the constructed nomogram can enhance patient management and facilitate individualized treatment plans. By using these indicators, clinicians can better stratify patients based on their prognosis and tailor their therapeutic approaches accordingly (Simon, 2010). Importantly, this model is specifically designed for preoperative use, enabling clinicians to assess patient prognosis and guide treatment decisions even before surgery is performed, without relying on intraoperative or postoperative data.

The biological relevance of each independent marker in the model further supports its robustness. Prolonged APTT suggests coagulation pathway disturbances, often related to impaired hepatic function or tumor-related coagulopathy, which may reflect a systemic pro-inflammatory state unfavorable for survival (Iba et al., 2025). Elevated GGT, a marker of oxidative stress and liver cell damage, has been associated with increased tumor burden and invasiveness (Jing et al., 2016). CA199, though classically used for pancreatic cancer, has been shown to reflect aggressive tumor biology and biliary obstruction in HCC(Mujica et al., 2000; Marrelli et al., 2009). CA125 elevation may indicate peritoneal irritation or advanced portal hypertension, indirectly reflecting tumor progression or liver decompensation (Chu et al., 2020). Finally, low PNR values may indicate neutrophil-driven tumor-promoting inflammation and immune suppression, both unfavorable prognostic signs (Bambace and Holmes, 2011; Horne et al., 2011; Gentles et al., 2015; Häuselmann et al., 2016; Lim et al., 2016; Nguyen et al., 2016; Khunger et al., 2018). Together, these markers capture liver function, tumor aggressiveness, and host systemic response, offering a more comprehensive reflection of patient status than TNM staging alone.

This study provides a more reliable prognostic tool for early HCC patients, which is expected to solve the prognosis assessment of early HCC patients in clinical practice, so as to help make more informed decisions in clinical practice.

When comparing our findings with existing literature, we observe both similarities and differences. Previous studies have established the prognostic role of markers such as NLR, PLR, and AFP in HCC (McMillan, 2009; Templeton et al., 2014b; Wang et al., 2019). However, few have specifically evaluated the role of APTT, CA199, CA125, and PNR in early-stage HCC prognosis. For instance, Jing et al. (Jing et al., 2020) suggested D-dimer as a prognostic marker, while our study demonstrates that CA199 and PNR—less commonly reported—have stronger prognostic implications when combined with TNM. Moreover, unlike many earlier studies that used a single marker, our model integrates multiple independent inflammatory and coagulation-related markers into a nomogram with external validation, offering greater predictive precision.

Nowadays, with further understanding of the biological behavior of liver cancer, the previous TNM is not comprehensive enough in evaluating the prognosis of HCC patients (Pascual et al., 2016). Combining TNM with serum biomarkers may be a new TNM model (Ding et al., 2024). The biological mechanisms underlying the prognostic impact of these preoperative serological inflammatory markers warrant further exploration. It is hypothesized that elevated levels of these markers may reflect systemic inflammation and immune response, which are known to influence tumor progression and patient outcomes (Ruiz-Ranz et al., 2022). For example, GGT and CA199 are associated with oxidative stress and chronic inflammation, which can promote carcinogenesis and metastasis (Li et al., 2019). Understanding these pathways could lead to targeted therapies that modulate these inflammatory responses, thereby improving prognosis and treatment efficacy for HCC patients (Refolo et al., 2020).

Compared with other studies, this study has advantages in sample size, rigorous study design, statistical analysis, etc. External data verification enhances the reliability of research conclusions. To further ensure model robustness, we also conducted internal validation using 1,000 bootstrap resamples, which helps reduce overfitting and supports the model’s stability. Although external validation data were not available in this study, we are actively planning future multicenter validation to confirm the model’s generalizability across broader clinical settings. However, certain limitations must be acknowledged. Potential selection bias, inherent to retrospective studies, could affect the generalizability of the findings. Additionally, the data sources may have limitations, such as incomplete records or variability in data quality. The follow-up period, while adequate, may not fully capture long-term outcomes, thus potentially underestimating late recurrence or survival rates (Antonarakis et al., 2012). Future studies should further investigate the biological roles of these markers using experimental and translational approaches, such as pathway analysis or immune profiling.

Further research should focus on multicenter studies to validate these findings in different populations and healthcare Settings (Wojcik et al., 2019). It will help to improve the accuracy and generalization of the prediction model. In addition, exploring new inflammatory markers or other prognostic factors may further understand the prognosis after HCC. Possible prospective studies or clinical trials to evaluate the clinical validity and usefulness of these predictive models in real-world Settings.

## 5 Conclusion

In this study, we developed and validated a prognostic nomogram for stage I/II HCC patients undergoing surgery, based on preoperative serological inflammatory markers combined with TNM staging. The nomogram, which incorporates APTT, GGT, CA199, CA125, and PNR, demonstrated good discrimination and calibration, and offers improved predictive performance over the traditional TNM system. These findings support the clinical utility of integrating systemic inflammatory markers into prognostic models, enabling more personalized postoperative management. Further multicenter validation and mechanistic studies are warranted.

## Data Availability

The raw data supporting the conclusions of this article will be made available by the authors, without undue reservation.
